# Mitochondrial metabolic remodeling predicts therapeutic response to PegIFN-α in chronic hepatitis B

**DOI:** 10.3389/fcimb.2025.1719456

**Published:** 2026-01-14

**Authors:** Yingying Zhang, Xiu Han, Chengyu Xu, Yahui Song, Jinghan Zhu, Ruoran Zhou, Yiling Chen, Mingming Liu, Junchi Xu, Xiangwei Wu, Qingzhen Han, Zutao Chen

**Affiliations:** 1 Department of Infectious Diseases, The Fourth Affiliated Hospital of Soochow University, Suzhou Dushu Lake Hospital, Suzhou, Jiangsu, China; 2 Center of Clinical Laboratory and Translational Medicine, The Fourth Affiliated Hospital of Soochow University, Suzhou Dushu Lake Hospital, Suzhou, Jiangsu, China; 3 Medical College of Soochow University, Suzhou, Jiangsu, China; 4 Center of Clinical Laboratory, The Fifth People’s Hospital of Suzhou, Suzhou, Jiangsu, China

**Keywords:** chronic hepatitis B, functional cure, immune exhaustion, mitochondrial metabolism, pegylated interferon-α

## Abstract

**Background:**

Chronic hepatitis B (CHB) remains a global health challenge, with current therapies achieving low rates of functional cure (FC). Reliable biomarkers are urgently needed to guide individualized treatment. This study characterized the immune–metabolic profiles of CHB patients receiving pegylated interferon-α (PegIFN-α) or nucleos(t)ide analogues (NAs), focusing on mitochondrial function as a novel predictor of therapeutic response.

**Methods:**

A total of 93 CHB patients and 32 healthy controls were recruited from three centers. Peripheral blood leukocyte subsets and mitochondrial parameters, including mitochondrial mass (MM) and the percentage of cells with low mitochondrial membrane potential (MMPlow%), were assessed by flow cytometry. Multivariate logistic regression and receiver operating characteristic (ROC) analyses were used to identify independent predictors and evaluate biomarker performance for FC.

**Results:**

Untreated CHB patients showed marked mitochondrial depletion across immune subsets. NA therapy normalized mitochondrial parameters without improving FC rates, whereas PegIFN-α therapy selectively remodeled CD4+ T cell metabolism and promoted monocyte differentiation. Improved mitochondrial efficiency in CD8+ T cells and elevated monocyte counts were closely associated with HBsAg clearance. Lymphocyte MMPlow% showed the strongest individual predictive value, while an integrated immune–metabolic model further enhanced accuracy for FC prediction.

**Conclusion:**

Immune–metabolic remodeling underlies PegIFN-α–induced functional cure in CHB. Mitochondrial profiling provides a promising framework for precision stratification and immune-based therapeutic optimization.

## 1 Introduction

Chronic hepatitis B (CHB) remains a major global health challenge despite effective viral suppression achieved by nucleos(t)ide analogues (NAs) (Hsu et al., 2023a). Functional cure (FC), defined as sustained hepatitis B surface antigen (HBsAg) clearance, remains rare and underscores the need for host-targeted therapeutic strategies. Current first-line therapies—NAs and pegylated interferon-α (PegIFN-α)—have distinct limitations (You et al., 2023). NAs efficiently inhibit HBV DNA polymerase but seldom induce HBsAg loss, necessitating lifelong therapy for most patients (Zoulim, 2004; Terrault et al., 2018; Hsu et al., 2023b; Tsai et al., 2024). In contrast, PegIFN-α acts through interferon-stimulated gene (ISG) activation, covalently closed circular DNA degradation, and immune modulation, and thus remains the only therapy capable of achieving FC (Yin et al., 2022; Jiang et al., 2024; Zhao et al., 2024). However, the rate of HBsAg clearance remains unsatisfactory, and reliable biomarkers to predict treatment response are lacking—posing a critical barrier to personalized therapy (European Association for the Study of the Liver, 2017).

Mitochondria have emerged as central regulators of immune cell fate by integrating metabolic and signaling cues to control T-cell activation, differentiation, and memory formation (van der Windt et al., 2012; Buck et al., 2016). In chronic viral infections, persistent antigen exposure triggers mitochondrial dysfunction and oxidative stress, driving T-cell exhaustion and loss of effector function (Bengsch et al., 2016; Fisicaro et al., 2020). Recent studies of chronic HBV infection have shown that HBV-specific CD8+ T cells exhibit defective glycolysis and mitochondrial respiration, linking metabolic insufficiency to immune failure (Schurich et al., 2016; Fisicaro et al., 2017; Winkler et al., 2023).

Emerging data further indicate that mitochondrial parameters—such as mitochondrial mass (MM) and mitochondrial membrane potential (MMP)—closely mirror immune competence. In untreated HBV infection, both MM and the proportion of cells with low MMP (MMPlow%) dynamically change with disease stage, paralleling T-cell exhaustion (Liu et al., 2025). Emerging evidence indicates that HBV infection actively disrupts mitochondrial dynamics rather than maintaining equilibrium. HBx-driven mitochondrial fission, dysfunction, and ROS accumulation impair antiviral signaling and contribute to immune exhaustion, providing a mechanistic basis for evaluating mitochondrial remodeling during therapy (Kim et al., 2013; Mansouri et al., 2018; Lin et al., 2024). These findings position mitochondrial dysfunction as a measurable hallmark of immune impairment. Yet, whether antiviral therapy—particularly PegIFN-α—can remodel mitochondrial metabolism and restore immune fitness remains unknown.

Building upon these insights, we systematically evaluated MM and MMPlow% in peripheral leukocyte subsets from CHB patients receiving PegIFN-α or NA therapy. Our study had two objectives: (i) to delineate PegIFN-α-induced mitochondrial remodeling in specific immune subsets, and (ii) to identify immune-metabolic biomarkers predictive of HBsAg clearance, addressing a critical unmet need in HBV therapeutics.

## 2 Methods

### 2.1 Study participants

Between July 2023 and April 2024, a total of 93 patients with CHB were recruited from three medical centers in Suzhou, Jiangsu Province, China: The Fourth Affiliated Hospital of Soochow University, The First Affiliated Hospital of Soochow University, and The Fifth People’s Hospital of Suzhou. Additionally, 32 healthy controls without a history of HBV infection or close contact were included. All CHB patients were aged 18 years or older and had been HBsAg- and HBV DNA-positive for more than 6 months prior to initiating NA or PegIFN-α therapy. Exclusion criteria included: (i) co-infection with other hepatitis viruses (A, C, or D) or human immunodeficiency virus; (ii) autoimmune liver diseases, alcoholic liver disease, decompensated cirrhosis, hepatocellular carcinoma, or other malignancies; and (iii) history of organ transplantation or long-term immunosuppressive therapy.

Healthy controls were confirmed to be disease-free through laboratory tests, imaging, and clinical assessment. CHB patients without prior antiviral treatment were classified as the untreated group. The PegIFN-α group included patients who had received ≥3 months of PegIFN-α therapy (135 μg or 180 μg), either as monotherapy or in combination with NA. The NA group comprised patients treated with NA monotherapy for more than 3 months. For both groups, peripheral blood samples were obtained during ongoing antiviral therapy, while all participants remained under active treatment at the time of collection—prior to therapy completion or endpoint evaluation. The study was conducted in accordance with the Declaration of Helsinki and was approved by the Institutional Review Boards of the participating hospitals (approval number: 241024). Written informed consent was obtained from all participants.

### 2.2 Assessment of serum virological markers and liver fibrosis

Serum HBV DNA levels were quantified using the Roche Cobas 4800 system with a real-time fluorescent quantitative PCR detection kit (Sansure Biotech, China), with a lower limit of detection of 60 IU/mL. Serum HBsAg and hepatitis B e antigen (HBeAg) concentrations were measured using commercial chemiluminescence immunoassay kits (Abbott, USA). The detection limit for HBsAg was 0.05 IU/mL, and HBeAg positivity was defined as >1 S/CO. Liver fibrosis was assessed noninvasively using transient elastography with FibroTouch and FibroScan devices according to the manufacturers’ instructions (EASL-ALEH Clinical Practice Guidelines, 2015; Xu et al., 2019).

### 2.3 Flow cytometric analysis of lymphocyte subsets and mitochondrial parameters

Lymphocyte subsets and mitochondrial parameters were assessed by flow cytometry using lymphocyte differentiation antigens (CD markers) and a mitochondrial-specific fluorescent probe (MitoDye, structural formula C_34_H_36_Cl_2_N_2_), as described previously (Ma et al., 2024; Zhou et al., 2024). The analyzed cell populations included granulocytes, monocytes, CD45+ lymphocytes, CD3+ T cells, CD3+CD4+CD8- T cells, CD3+CD4-CD8+ T cells, CD4+CD8+ double-positive T cells, CD4-CD8- T cells, CD3-CD19+ B cells, and CD3-CD56+ natural killer (NK) cells. MM and MMPlow% were quantified for each subset. Flow cytometry data were analyzed using a standardized gating strategy (**Supplementary Figure S1**).

### 2.4 Statistical analysis

Statistical analyses were performed using GraphPad Prism version 10.0 and SPSS version 26.0. Continuous variables with normal distribution and homogeneous variance were expressed as mean ± standard deviation (SD); non-normally distributed data were presented as median (interquartile range [IQR]). For comparisons between two groups, the independent samples t-test or the Mann-Whitney U test was used, depending on data distribution. Categorical variables were analyzed using the chi-square test. For comparisons among three or more groups, one-way analysis of variance (ANOVA) followed by Dunnett’s *post hoc* test was used, with the healthy control group as the reference.

Variables with P < 0.05 in univariate analysis were entered into multivariate logistic regression to identify independent predictors of HBsAg clearance. Receiver operating characteristic (ROC) curve analyses were used to evaluate the predictive performance of both individual biomarkers and the combined model derived from multivariate logistic regression. The area under the curve (AUC), 95% confidence interval (CI), optimal cut-off (Youden index), sensitivity, and specificity were calculated. Model robustness was assessed using five-fold cross-validation, with mean AUC ± standard error (SE) values averaged across folds. All tests were two-tailed, and P < 0.05 was considered statistically significant.

## 3 Results

### 3.1 Participant characteristics

A total of 93 patients with CHB and 32 healthy controls were enrolled in this study. Patients with CHB were stratified into untreated (n = 18), PegIFN-α–treated (n = 42; monotherapy, n = 4; combination with NA, n = 38), and NA-monotherapy (n = 33; entecavir, n = 5; tenofovir disoproxil fumarate, n = 1; tenofovir alafenamide, n = 3; tenofovir amibufenamide, n = 24) groups (**Table 1**). Age distributions differed significantly among the four groups (P = 0.021), primarily due to differences between the untreated and PegIFN-α groups. Sex ratios were comparable across groups (P = 0.253). Rates of HBV DNA and HBsAg positivity varied markedly among CHB subgroups (P < 0.001 and P = 0.005, respectively), whereas HBeAg positivity rates were similar (P = 0.318). Fibrosis stages, assessed by transient elastography, showed no significant differences (P = 0.820), and no participants had cirrhosis or hepatocellular carcinoma.

**Table 1:** Baseline demographic and clinical characteristics of study participants by group.

Variables	Healthy control (n=32)	Untreated group (n=18)	PegIFN-α group (n=42)	NA group (n=33)	*P* value
Age, years	36 (13)	34 (9)	45 (18)	38 (21)	0.021
Male (%)	59.38	44.44	71.43	63.64	0.253
Course of hepatitis B disease, n (%)					0.314
<10 years	/	4 (22.22)	18 (42.86)	12 (36.36)	
≥10 years	/	14 (77.78)	24 (57.14)	21 (63.64)	
HBV DNA positivity, n (%)	/	18 (100)	2 (4.76)	8 (24.24)	<0.001
HBsAg positivity, n (%)	/	18 (100)	32 (76.19)	32 (96.97)	0.005
HBeAg positivity, n (%)	/	5 (27.78)	5 (11.90)	7 (21.21)	0.318
Liver fibrosis grading, n(%)					0.820
no liver fibrosis	/	16 (88.89)	35 (83.33)	30 (90.91)	
mild liver fibrosis	/	1 (5.56)	4 (9.52)	3 (9.09)	
moderate liver fibrosis	/	1 (5.56)	2 (4.76)	0	
severe liver fibrosis	/	0	1 (2.38)	0	

### 3.2 Peripheral blood leukocyte profiles and mitochondrial features in CHB subgroups

Peripheral blood immune profiles and mitochondrial parameters were assessed during ongoing antiviral therapy in treated CHB patients, and at enrollment in untreated patients and healthy controls (**Table 2**). All CHB subgroups exhibited reduced total lymphocyte counts compared to controls (P < 0.001), with the most pronounced reductions in the PegIFN-α group, affecting total lymphocytes, CD3+ T cells, CD4+ T cells, and CD4+CD8+ double-positive T cells. B cell proportions were highest in untreated CHB patients (P = 0.004), whereas monocyte percentages were elevated in PegIFN-α–treated patients (P < 0.001), who also showed the lowest CD4+ T cell percentages (P = 0.002).

**Table 2:** Comparison of peripheral blood immune cell and mitochondrial function in different populations.

Variables	Healthy control (n=32)	Untreated group (n=18)	PegIFN-α group (n=42)	NA group (n=33)	*P* value
Granulocyte count (/μL)	3031.21 (2015.39)	2068.28 (2787.14)	2153.50 (1478.81)	2976.88 (1617.44)	0.068
Granulocyte percentage (%)	50.93 ± 12.06	52.90 ± 10.58	54.42 (15.58)	55.00 (14.02)	0.363
Granulocyte MMPlow%	18.12 (29.22)	13.65 (24.27)	17.52 (30.72)	13.29 (12.61)	0.329
Granulocyte MM	3.79 (3.35)	1.32 (0.80) ↓↓↓	2.31 (1.75)	3.42 (2.80)	<0.001
Lymphocyte count (/μL)	2054.39 (1377.03)	1736.18 (994.59)	1293.50 (590.67) ↓↓	1759.26 (757.61)	<0.001
Lymphocyte percentage (%)	37.85 ± 12.86	33.72 ± 10.28	30.39 (17.28)	34.12 (12.32)	0.596
Lymphocyte MMPlow%	49.97 ± 17.41	59.56 ± 20.55	41.62 (29.83)	43.46 (28.73)	0.015
Lymphocyte MM	1.44 (1.25)	0.75 (0.64) ↓↓	1.29 (0.70)	1.55 (0.81)	0.005
Monocyte count (/μL)	384.19 (262.96)	346.17 (337.66)	359.42 (176.82)	315.49 (174.60)	0.187
Monocyte percentage (%)	6.60 (2.57)	6.27 (2.81)	9.08 (4.26) ↑↑	5.84 (2.70)	<0.001
Monocyte MMPlow%	17.74 (40.34)	12.55 (32.18)	21.00 (26.55)	15.92 (20.95)	0.158
Monocyte MM	6.06 (7.04)	1.95 (2.53) ↓	2.55 (1.82) ↓↓	3.30 (3.90)	<0.001
CD3+ T cell count (/μL)	1407.61 (1080.45)	1163.01 (721.95)	800.28 (515.84) ↓↓	1229.91 (541.52)	<0.001
CD3+ T cell percentage (%)	68.42 ± 10.64	67.93 ± 6.73	64.32 ± 11.35	68.78 ± 9.82	0.206
CD3+ T cell MMPlow%	63.66 (22.31)	65.33 (25.63)	70.46 (19.68)	61.66 (22.85)	0.733
CD3+ T cell MM	1.42 (0.96)	0.78 (0.50) ↓↓↓	1.12 ± 0.53	1.43 ± 0.53	<0.001
CD3+CD4+ T cell count (/μL)	621.16 (501.24)	531.22 (414.19)	290.41 (230.91) ↓↓↓	510.93 (260.22)	<0.001
CD3+CD4+ T cell percentage (%)	32.69 ± 8.93	33.18 ± 8.69	26.14 ± 8.39 ↓	31.18 ± 7.66	0.002
CD3+CD4+ T cell MMPlow%	65.90 (17.52)	66.55 (30.71)	77.62 (22.40) ↑	69.21 (18.65)	0.007
CD3+CD4+ T cell MM	1.51 (0.96)	0.80 (0.48) ↓↓↓	1.05 (0.82) ↓↓	1.44 (0.78)	<0.001
CD3+CD8+ T cell count (/μL)	515.34 (437.94)	474.84 (164.46)	404.80 (397.95)	474.12 (303.76)	0.020
CD3+CD8+ T cell percentage (%)	24.44 (12.11)	25.61 (9.21)	29.14 ± 11.98	25.94 ± 8.11	0.597
CD3+CD8+ T cell MMPlow%	63.56 ± 15.76	59.59 ± 19.19	65.42 (18.16)	62.45 (24.75)	0.851
CD3+CD8+ T cell MM	1.27 (0.81)	0.72 (0.57) ↓↓	1.22 ± 0.61	1.50 ± 0.59	0.004
CD4+CD8+ T cell count (/μL)	14.82 (26.96)	14.52 (17.33)	5.55 (10.17) ↓	12.03 (22.24)	0.012
CD4+CD8+ T cell percentage (%)	0.81 (0.71)	0.84 (0.80)	0.41 (0.79)	0.95 (1.02)	0.469
CD4+CD8+ T cell MMPlow%	54.20 ± 16.56	52.25 ± 22.26	54.33 ± 19.11	54.20 ± 16.55	0.980
CD4+CD8+ T cell MM	1.74 (1.17)	0.93 (0.59) ↓↓	1.56 (1.14)	1.73 (1.02)	0.002
CD4-CD8- T cell count (/μL)	133.64 (160.34)	157.76 (88.17)	78.65 (120.83)	155.83 (264.53)	0.004
CD4-CD8- T cell percentage (%)	6.46 (8.02)	8.37 (4.21)	9.33 (6.01)	9.73 (10.54)	0.056
CD4-CD8- T cell MMPlow%	69.53 ± 14.47	62.21 ± 19.72	72.30 (23.05)	68.32 (24.01)	0.262
CD4-CD8- T cell MM	1.23 (0.59)	0.75 (0.26) ↓↓↓	1.10 (0.80)	1.36 (0.80)	0.001
CD19+ B cell count (/μL)	161.88 (165.39)	216.11 (167.58)	112.53 (73.19)	123.96 (114.55)	0.001
CD19+ B cell percentage (%)	7.92 (4.70)	11.64 (6.79)	9.46 (6.42)	7.90 (3.47)	0.004
CD19+ B cell MMPlow%	67.11 ± 19.34	65.07 ± 18.60	68.04 ± 14.93	63.53 ± 16.42	0.691
CD19+ B cell MM	1.54 (2.16)	0.72 (0.62) ↓↓	1.09 (0.63)	1.41 (1.75)	<0.001
CD56+ NK cell count (/μL)	391.63 (302.58)	330.04 (128.90)	284.46 (234.74)	360.23 (234.57)	0.010
CD56+ NK cell percentage (%)	22.30 ± 9.51	18.73 ± 7.33	24.81 ± 9.52	22.95 ± 10.25	0.155
CD56+ NK cell MMPlow%	69.03 ± 12.94	63.48 ± 17.34	65.04 (29.57)	61.56 (20.02)	0.155
CD56+ NK cell MM	1.77 (1.36)	0.74 (0.54) ↓↓	1.23 (0.84)	1.92 (1.15)	0.001

The symbols in the table indicate statistical comparisons of different subgroups of HBV infection with the healthy control group: ↑ denotes a significant increase (P < 0.05); ↓ indicates a significant decrease (P < 0.05); ↓↓ represents a more significant decrease (P < 0.01); ↓↓↓ indicates the most significant decrease (P < 0.001).

Untreated CHB patients displayed significant reductions in MM across all leukocyte subsets relative to healthy controls (P < 0.001). During ongoing NA therapy, MM and MMPlow% levels in leukocyte subsets were maintained within the normal range, comparable to those of healthy controls, reflecting metabolic stabilization without major immune remodeling. In contrast, PegIFN-α therapy induced distinctive metabolic reprogramming, characterized by decreased MM (P < 0.001) and increased MMPlow% (P = 0.007) specifically in CD3+CD4+ T cells—a pattern associated with subsequent HBsAg clearance in FC-achievers.

### 3.3 Univariate analysis of immune correlates of HBsAg clearance during PegIFN-α therapy

To identify immune and mitochondrial correlates associated with treatment response, patients receiving PegIFN-α were stratified according to HBsAg clearance status. HBsAg clearers had significantly higher monocyte counts (P = 0.004) and CD4-CD8- T cell counts (P = 0.008), but lower B cell percentages (P = 0.011), compared to non-clearers. Additionally, HBsAg clearers exhibited lower MMPlow% in total lymphocytes (P = 0.003), CD3+ T cells (P = 0.024), CD4+ T cells (P = 0.005), and CD8+ T cells (P = 0.026) (**Table 3**). Variables with P < 0.05 in univariate analysis were subsequently included in multivariate logistic regression to identify independent predictors of FC (**Table 4**). No significant difference in baseline HBV DNA levels was observed between FC and non-FC patients (P > 0.05), and HBV DNA was therefore not considered a discriminative variable for subsequent multivariate modeling.

**Table 3:** Comparison of immune and mitochondrial markers between HBsAg clearers and non-clearers in PegIFN-α-treated patients.

Variables	HBsAg-negative group (n=10)	HBsAg-positive group (n = 32)	*P* value
Granulocyte count (/μL)	3036.89 (1631.83)	2064.22 (1195.24)	0.137
Granulocyte percentage (%)	53.75 (11.04)	54.42 (15.58)	0.894
Granulocyte MMPlow%	17.80 (15.95)	17.52 (36.78)	0.281
Granulocyte MM	2.53 (0.78)	2.14 (1.95)	0.392
Lymphocyte count (/μL)	1556.49 (616.82)	1151.97 (519.00)	0.108
Lymphocyte percentage (%)	24.55 (9.21)	35.44 (19.58)	0.009
Lymphocyte MMPlow%	53.93 (29.02)	39.66 (19.65)	0.003
Lymphocyte MM	1.27 (0.50)	1.29 (0.73)	0.813
Monocyte count (μ/L)	645.85 (526.91)	326.38 (145.07)	0.004
Monocyte percentage (%)	11.55 (6.80)	8.96 (3.09)	0.231
Monocyte MMPlow%	30.47 (19.73)	18.41 (21.41)	0.048
Monocyte MM	2.85 (0.80)	2.40 (1.91)	0.256
CD3+ T cell count (/μL)	1094.16 (390.48)	696.73 (441.39)	0.096
CD3+ T cell percentage (%)	67.32 (17.19)	65.92 (13.89)	0.712
CD3+ T cell MMPlow%	53.08 (30.18)	71.64 (16.21)	0.024
CD3+ T cell MM	1.33 (0.36)	1.11 (0.97)	0.089
CD3+CD4+ T cell count (/μL)	349.52 (312.73)	285.08 (195.84)	0.494
CD3+CD4+ T cell percentage (%)	28.84 (15.85)	25.30 (11.48)	0.942
CD3+CD4+ T cell MMPlow%	63.70 (20.36)	81.24 (20.22)	0.005
CD3+CD4+ T cell MM	1.20 (0.33)	0.99 (0.79)	0.174
CD3+CD8+ T cell count (/μL)	582.34 (319.23)	291.56 (277.27)	0.064
CD3+CD8+ T cell percentage (%)	36.84 (6.26)	25.52 (15.79)	0.213
CD3+CD8+ T cell MMPlow%	54.39 (24.55)	66.50 (17.61)	0.026
CD3+CD8+ T cell MM	1.31 (0.48)	0.99 (0.79)	0.062
CD4+CD8+ T cell count (/μL)	9.46 (27.69)	5.08 (8.57)	0.152
CD4+CD8+ T cell percentage (%)	0.45 (1.40)	0.39 (0.72)	0.243
CD4+CD8+ T cell MMPlow%	52.27 (19.55)	55.00 (33.27)	0.469
CD4+CD8+ T cell MM	1.60 (0.28)	1.54 (1.37)	0.565
CD4-CD8- T cell count (/μL)	185.75 (160.74)	69.50 (120.95)	0.008
CD4-CD8- T cell percentage (%)	10.64 (7.04)	8.43 (5.33)	0.308
CD4-CD8- T cell MMPlow%	67.25 (31.14)	72.79 (19.92)	0.128
CD4-CD8- T cell MM	1.23 (0.53)	1.01 (0.77)	0.204
CD19+ B cell count (/μL)	91.41 (83.20)	115.33 (71.05)	0.172
CD19+ B cell percentage (%)	6.46 (1.86)	10.16 (7.53)	0.011
CD19+ B cell MMPlow%	65.88 (21.70)	71.83 (18.08)	0.064
CD19+ B cell MM	1.22 (0.39)	0.96 (0.68)	0.132
CD56+ NK cell count (/μL)	379.07 (269.04)	282.71 (160.96)	0.224
CD56+ NK cell percentage (%)	25.09 (15.11)	23.07 (12.31)	0.673
CD56+ NK cell MMPlow%	65.69 (14.17)	65.03 (29.68)	0.805
CD56+ NK cell MM	1.20 (0.46)	1.23 (1.15)	1.000

**Table 4:** Multivariate logistic regression analysis of immune parameters associated with HBsAg clearance in patients treated with PegIFN-α.

Variable	*β* coefficient	Odds Ratio	95% Confidence interval	*P* value
Monocyte count (μ/L)	–0.006	0.994	0.989 - 0.999	0.014
CD4-CD8- T cell count (/μL)	–0.012	0.988	0.979 - 0.997	0.008
Lymphocyte percentage (%)	0.084	1.088	0.998 - 1.185	0.055
CD19+ B cell percentage (%)	0.275	1.316	1.031 - 1.680	0.027
Lymphocyte MMPlow%	–0.065	0.937	0.892 - 0.984	0.009
Monocyte MMPlow%	–0.041	0.960	0.917 - 1.005	0.080
CD3+ T cell MMPlow%	0.052	1.054	1.007 - 1.103	0.025
CD3+CD4+ T cell MMPlow%	0.058	1.059	1.009 - 1.113	0.021
CD3+CD8+ T cell MMPlow%	0.046	1.048	1.002 - 1.095	0.039

### 3.4 Multivariate analysis of predictors for HBsAg clearance during PegIFN-α therapy

Multivariate logistic regression incorporating immune and metabolic variables identified seven parameters independently associated with functional cure (**Table 4**). Among these, monocyte count (β = –0.006, P = 0.014), CD4^-^CD8^-^ T-cell count (β = –0.012, P = 0.008), B-cell percentage (β = 0.275, P = 0.027), and lymphocyte MMPlow% (β = –0.065, P = 0.009) showed the strongest associations. These findings provided the statistical foundation for subsequent evaluation of predictive performance.

### 3.5 Predictive performance of immune-metabolic biomarkers for FC

**Figure 1:**
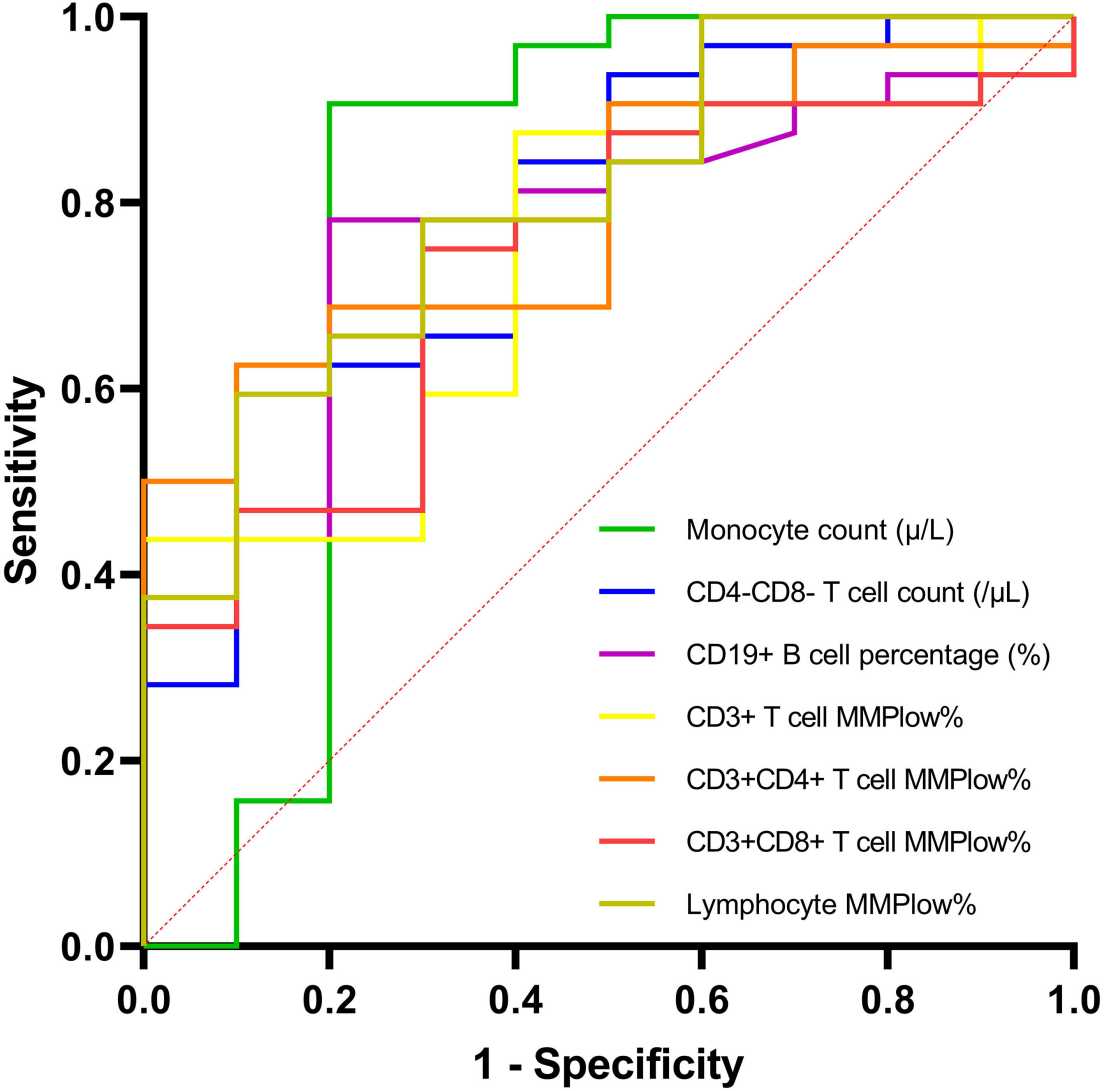
ROC curves for selected immune and mitochondrial biomarkers predicting FC in PegIFN-α–treated patients.

Based on these independent predictors, ROC analyses (**Table 5**, **Figure 1**) showed that lymphocyte MMPlow% achieved the highest discriminative ability (AUC = 0.803), followed by monocyte count (AUC = 0.794), CD4-CD8- T cell count (AUC = 0.775), CD4+ T cell MMPlow% (AUC = 0.791), and B cell percentage (AUC = 0.770). These immune–metabolic biomarkers displayed moderate-to-high accuracy in predicting functional cure among PegIFN-α–treated CHB patients. Five-fold cross-validation yielded consistent AUC values across folds, confirming the robustness of the predictive performance (**Table 5**).

**Table 5:** ROC analysis of immune markers predicting HBsAg clearance in PegIFN-α–treated patients.

Prediction model	AUC ± SE	Cut-off point	Sensitivity	Specificity	*P* value
Monocyte count (/μL)	0.794 ± 0.121	426.00	90.62%	80%	0.016
CD4-CD8- T cell count (/μL)	0.775 ± 0.089	151.17	84.37%	60%	0.002
CD19+ B cell percentage (%)	0.770 ± 0.082	7.90	78.12%	80%	0.0009
Lymphocyte MMPlow%	0.803 ± 0.078	41.39	59.38%	90%	0.0001
CD3+ T cell MMPlow%	0.738 ± 0.089	53.60	87.50%	60%	0.007
CD3+CD4+ T cell MMPlow%	0.791 ± 0.074	77.46	62.50%	90%	0.0001
CD3+CD8+ T cell MMPlow%	0.734 ± 0.087	56.80	75.00%	70%	0.007

**Figure 2:**
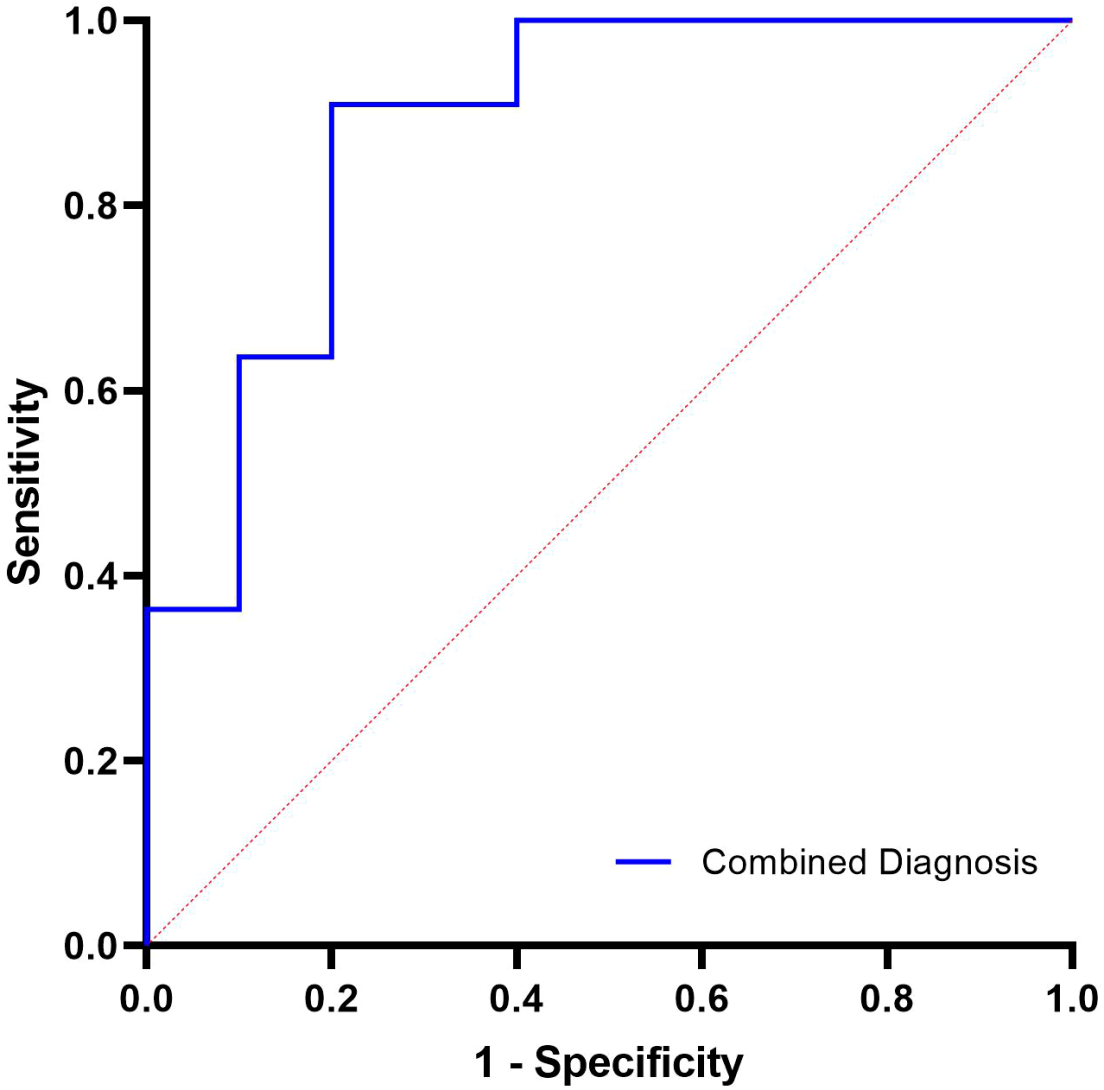
ROC curve of the combined immune-metabolic model.

Variables included in the combined model were selected based on independent significance in multivariate logistic regression (**Table 4**) and complementary biological relevance. Monocyte count represents innate immune activation, B-cell percentage reflects adaptive immune coordination, and lymphocyte MMPlow% captures mitochondrial metabolic fitness. A logistic-regression–based combined model incorporating these three parameters was then established. As shown in **Figure 2**, the model achieved an AUC of 0.882 (95% CI 0.667-0.980, P < 0.0001), with an optimal cut-off of 0.388 yielding 90.91% sensitivity and 80.00% specificity. This performance exceeded that of any single biomarker, underscoring the additive predictive value of integrating innate, adaptive, and metabolic features for identifying PegIFN-α responders.

## 4 Discussion

**Figure 3:**
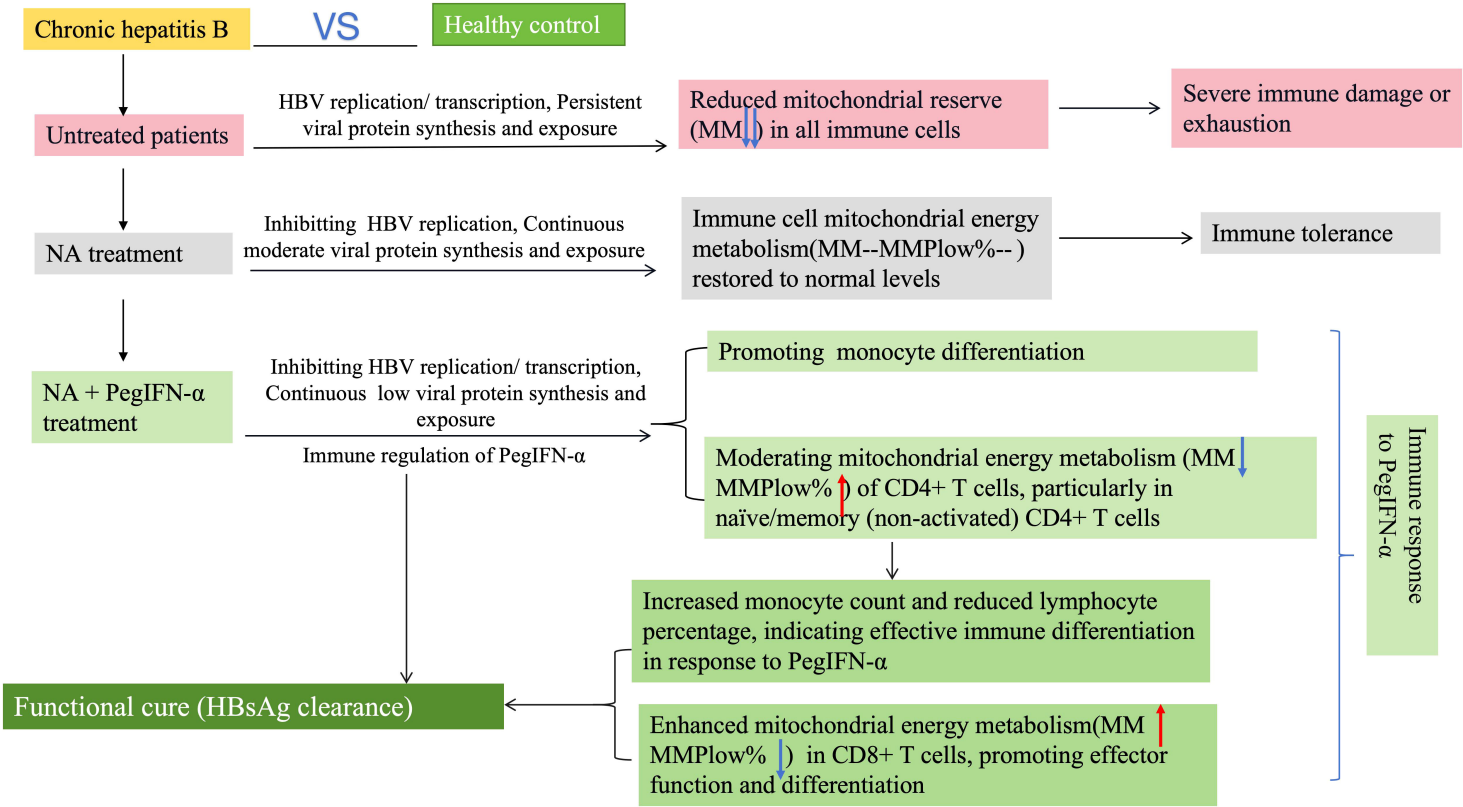
Immune and mitochondrial features associated with antiviral strategies in CHB.

This study provides a comprehensive immune-metabolic characterization of CHB patients receiving PegIFN-α or NAs, revealing mitochondrial dysfunction as a central feature of immune exhaustion and identifying novel biomarkers for FC. We demonstrate that untreated CHB induces global mitochondrial depletion across leukocyte subsets, while PegIFN-α—but not NA—selectively remodels CD4+ T cell metabolism and promotes monocyte differentiation, with these changes strongly correlating with HBsAg clearance. Notably, lymphocyte MMPlow% achieved an AUC of 0.803 in predicting FC, outperforming conventional immune markers and offering a potentially actionable tool for personalized therapy in alignment with WHO’s 2030 hepatitis elimination goals. A graphical summary of these immune-metabolic pathways and therapeutic outcomes is illustrated in **Figure 3**. Our findings are consistent with recent evidence linking mitochondrial remodeling to immune exhaustion in chronic HBV infection. In untreated patients, MM increases and MMPlow% declines progressively from CHB to cirrhosis and hepatocellular carcinoma, reflecting compensatory mitochondrial expansion amid functional decline (Liu et al., 2025). In contrast, in our PegIFN-α–treated cohort, FC achievers exhibited reduced MMPlow% and improved mitochondrial efficiency, indicating partial reversal of this exhaustion-associated phenotype. Together, these data suggest that mitochondrial parameters not only reflect immune dysfunction but also capture the dynamic process of immune restoration under antiviral therapy. These findings are consistent with established evidence that HBV infection disrupts mitochondrial homeostasis. HBx has been shown to promote excessive mitochondrial fission, alter the balance of fusion–fission regulators, and induce mitophagy and loss of membrane potential, thereby contributing to metabolic insufficiency and impaired antiviral immunity (Kim et al., 2013; Mansouri et al., 2018; Lin et al., 2024). The mitochondrial remodeling observed in our PegIFN-α cohort may therefore reflect, at least in part, the reversal of HBV-induced mitochondrial dysregulation.

Importantly, all treated patients were still under ongoing PegIFN-α or NA therapy at the time of sampling, indicating that immune-metabolic remodeling occurred during active treatment rather than after its completion. This ensures that the observed mitochondrial and immune signatures reflect on-treatment processes potentially predictive of subsequent functional cure, rather than post-therapy correlates. The profound reduction in MM in untreated CHB patients underscores the systemic impact of persistent HBV replication on immune cell energetics. This aligns with prior work linking chronic antigen exposure to T cell exhaustion via oxidative stress and metabolic insufficiency (Hu et al., 2011; Fisicaro et al., 2020). While NA therapy normalized MM and MMPlow% to healthy control levels—consistent with its role in suppressing viral replication without immune modulation—it failed to restore CD8+ T cell function or achieve FC in most patients (Liu et al., 2019; Tout et al., 2020). In contrast, PegIFN-α uniquely induced a divergent metabolic response: reduced MM and elevated MMPlow% in CD4+ T cells, a pattern that may reflect broader immunometabolic effects of type I interferon signaling. PegIFN-α induces a strong ISG response, and prior studies have shown that sustained inflammatory and interferon-rich environments in chronic viral infection can influence mitochondrial homeostasis and membrane potential (Li and Ou, 2023; Su et al., 2024). These established observations provide a biological context for the mitochondrial phenotype we observed in CD4+ T cells during PegIFN-α therapy, although the precise mechanisms underlying this remodeling remain to be fully elucidated. Importantly, this CD4+ T-cell phenotype contrasts with the mitochondrial improvements observed in CD8+ T cells among HBsAg clearers, who demonstrated markedly lower MMPlow%—a profile indicative of enhanced mitochondrial efficiency. This finding is consistent with previous work showing that restoration of mitochondrial fitness is a key step in reversing exhaustion-associated metabolic defects in HBV-specific CD8+ T cells (Schurich et al., 2016; Fisicaro et al., 2017; Winkler et al., 2023). Taken together, these divergent remodeling patterns—interferon-associated mitochondrial modulation in CD4+ T cells and restoration of mitochondrial competence in CD8+ T cells—may represent complementary aspects of PegIFN-α–driven immune reconditioning that support FC. Although HBV DNA positivity rates differed significantly between groups, high viral load is known to drive T-cell exhaustion through sustained inhibitory receptor upregulation (Ye et al., 2015). However, once HBV DNA exceeds ~10^5^ copies/mL, further increases correlate only weakly with intrahepatic inflammation or fibrosis (Shao et al., 2007). In our cohort, FC achievers and non-achievers had comparable baseline HBV DNA, yet showed striking mitochondrial differences. Thus, the immune-metabolic remodeling we observed appears primarily driven by treatment-specific immune activation rather than by baseline viral load alone.

Multivariate analysis identified seven independent predictors of FC, with monocyte count, CD4-CD8- T cell count, B-cell percentage, and lymphocyte MMPlow% showing the strongest associations, highlighting the interplay between innate and adaptive immunity in viral clearance. CD4-CD8- T cells, although representing a relatively small peripheral subset, comprise heterogeneous populations including γδ T cells and unconventional αβ T cells. These innate-like T cells are capable of rapid cytokine secretion and non-classical cytotoxic responses during viral infection, and their enrichment in FC achievers suggests that PegIFN-α may enhance antiviral immunity beyond conventional CD4+ and CD8+ T-cell pathways. While our cross-sectional design does not allow mechanistic confirmation, the association between higher CD4-CD8- T cell levels and FC is consistent with the broader concept that multiple T-cell compartments—including innate-like subsets—cooperate in HBV immune control. Elevated monocytes in FC achievers likely reflect PegIFN-α–associated immune activation, potentially enhancing antigen presentation and supporting coordinated T-cell responses (Mehrotra et al., 2020; Jiang et al., 2024), complementing the contribution of CD4-CD8- T cells to antiviral immunity. ROC analysis demonstrated that lymphocyte MMPlow% achieved the highest discriminative ability (AUC = 0.803), and a combined model integrating monocyte count, B-cell percentage, and lymphocyte MMPlow% further improved predictive performance (AUC = 0.882). In contrast, traditional virological and serological markers—such as baseline HBsAg, HBV DNA, and anti-HBc—have shown only moderate predictive accuracy for PegIFN-α response. Meta-analytic data indicate pooled AUCs below 0.70 for baseline HBsAg and HBV DNA, while a multicenter study reported week-12 anti-HBc levels predicting HBsAg clearance with AUCs of 0.61–0.74 (Jiang et al., 2024; Wang et al., 2024). Even newer viral markers such as HBcrAg exhibit better but still modest performance, with lower baseline HBcrAg associated with higher spontaneous HBsAg seroclearance (adjusted HR ≈ 1.9) (Tseng et al., 2023). Compared with conventional clinical predictors—including HBsAg, anti-HBc, and HBcrAg—which typically achieve only modest discrimination for PegIFN-α response, the immune-metabolic markers identified in this study provide substantially stronger predictive performance. These findings highlight the added value of incorporating mitochondrial metrics into response assessment algorithms. Future work should include longitudinal metabolic tracking and single-cell mechanistic analyses to further define how PegIFN-α reshapes immune function over the course of therapy.

Clinically, our findings advocate for integrating immune-metabolic profiling into CHB management. The association between mitochondrial integrity and FC suggests therapeutic potential for metabolic modulators (such as mitophagy enhancers or OXPHOS promoters) to synergize with PegIFN-α, an approach supported by preclinical models (Fisicaro et al., 2017). Additionally, ROC-optimized biomarker panels (for example, MMPlow% plus monocyte count) could guide trial design for novel FC-targeting regimens, such as PegIFN-α/NA combinations or checkpoint inhibitors.

To strengthen the internal validity of our predictive model, we implemented a five-fold cross-validation approach, which confirmed the stability of AUC estimates and reduced potential overfitting bias. While our multicenter design strengthens generalizability, larger cohorts are needed to validate rare subset analyses (for example, CD4-CD8- T cells). The cross-sectional sampling limits causal inference; longitudinal studies tracking mitochondrial dynamics during therapy will clarify kinetic relationships. Finally, mechanistic studies—using single-cell RNA sequencing or mitochondrial flux assays—should dissect how PegIFN-α restores T-cell metabolism and whether these pathways can be pharmacologically harnessed. Future studies with larger prospective cohorts and complementary mechanistic models—such as animal studies or ex vivo functional assays—will be essential to further validate these immune–metabolic biomarkers and elucidate the mitochondrial pathways underlying PegIFN-α responsiveness.

In summary, our study establishes mitochondrial dysfunction as a hallmark of CHB-induced immune exhaustion and defines immune-metabolic signatures that robustly predict the efficacy of PegIFN-α therapy. These insights bridge virology, immunology, and metabolism, providing both biomarker-driven strategies for current therapies and new targets for curative interventions.

## Data Availability

The raw data supporting the conclusions of this article will be made available by the authors, without undue reservation.

## Supplementary material

Supplementary Figure 1 Manual gating strategy. Figure shows the gating strategy for lymphocyte subsets and mitochondrial parameters (MM and MMPlow%) by flow cytometry. The CD3 antibody is in the same lane (PE) as the CD56 antibody but has a different level of fluorescence intensity, which allows us to specifically recognize cell populations. The same forms of CD19 and CD8 are the same. The main population of cells can be identified based on different expressions, and the aim is to use fewer channels to achieve multi-parameter detection (patent number: CN 112098646 B). The Mito APC-H channel shows the mitochondrial MMP clustering of the corresponding cell population.

## References

[B1] Bengsch B. Johnson A. L. Kurachi M. Odorizzi P. M. Pauken K. E. Attanasio J. . (2016). Bioenergetic insufficiencies due to metabolic alterations regulated by the inhibitory receptor PD-1 are an early driver of CD8(+) T cell exhaustion. Immunity 45, 358–373. doi: 10.1016/j.immuni.2016.07.008, PMID: 27496729 PMC4988919

[B2] Buck M. D. O’Sullivan D. Klein Geltink R. I. Curtis J. D. Chang C. H. Sanin D. E. . (2016). Mitochondrial dynamics controls T cell fate through metabolic programming. Cell 166, 63–76. doi: 10.1016/j.cell.2016.05.035, PMID: 27293185 PMC4974356

[B3] EASL-ALEH Clinical Practice Guidelines (2015). Non-invasive tests for evaluation of liver disease severity and prognosis. J. Hepatol. 63, 237–264. doi: 10.1016/j.jhep.2015.04.006, PMID: 25911335

[B4] European Association for the Study of the Liver (2017). Electronic address eee, European Association for the Study of the L. EASL 2017 Clinical Practice Guidelines on the management of hepatitis B virus infection. J. Hepatol. 67, 370–398. doi: 10.1016/j.jhep.2017.03.021, PMID: 28427875

[B5] Fisicaro P. Barili V. Montanini B. Acerbi G. Ferracin M. Guerrieri F. . (2017). Targeting mitochondrial dysfunction can restore antiviral activity of exhausted HBV-specific CD8 T cells in chronic hepatitis B. Nat. Med. 23, 327–336. doi: 10.1038/nm.4275, PMID: 28165481

[B6] Fisicaro P. Barili V. Rossi M. Montali I. Vecchi A. Acerbi G. . (2020). Pathogenetic mechanisms of T cell dysfunction in chronic HBV infection and related therapeutic approaches. Front. Immunol. 11, 849. doi: 10.3389/fimmu.2020.00849, PMID: 32477347 PMC7235343

[B7] Hsu Y. C. Huang D. Q. Nguyen M. H. (2023a). Global burden of hepatitis B virus: current status, missed opportunities and a call for action. Nat. Rev. Gastroenterol. Hepatol. 20, 524–537. doi: 10.1038/s41575-023-00760-9, PMID: 37024566

[B8] Hsu Y. C. Tseng C. H. Kao J. H. (2023b). Safety considerations for withdrawal of nucleos(t)ide analogues in patients with chronic hepatitis B: First, do no harm. Clin. Mol. Hepatol. 29, 869–890. doi: 10.3350/cmh.2022.0420, PMID: 36916171 PMC10577354

[B9] Hu L. Chen L. Yang G. Li L. Sun H. Chang Y. . (2011). HBx sensitizes cells to oxidative stress-induced apoptosis by accelerating the loss of Mcl-1 protein via caspase-3 cascade. Mol. Cancer 10, 43. doi: 10.1186/1476-4598-10-43, PMID: 21504623 PMC3096594

[B10] Jiang P. Jia H. Qian X. Tang T. Han Y. Zhang Z. . (2024). Single-cell RNA sequencing reveals the immunoregulatory roles of PegIFN-α in patients with chronic hepatitis B. Hepatology 79, 167–182. doi: 10.1097/HEP.0000000000000524, PMID: 37368993

[B11] Jiang S. Guo S. Huang Y. Yin Y. Feng J. Zhou H. . (2024). Predictors of HBsAg seroclearance in patients with chronic HBV infection treated with pegylated interferon-α: a systematic review and meta-analysis. Hepatol. Int. 18, 892–903. doi: 10.1007/s12072-024-10648-8, PMID: 38461186 PMC11126512

[B12] Kim S. J. Khan M. Quan J. Till A. Subramani S. Siddiqui A. (2013). Hepatitis B virus disrupts mitochondrial dynamics: induces fission and mitophagy to attenuate apoptosis. PloS Pathog. 9, e1003722. doi: 10.1371/journal.ppat.1003722, PMID: 24339771 PMC3855539

[B13] Li Y. Ou J. J. (2023). Regulation of mitochondrial metabolism by hepatitis B virus. Viruses 15, 1–12. doi: 10.3390/v15122359, PMID: 38140600 PMC10747323

[B14] Lin C. Luo L. Xun Z. Zhu C. Huang Y. Ye Y. . (2024). Novel function of MOTS-c in mitochondrial remodelling contributes to its antiviral role during HBV infection. Gut 73, 338–349. doi: 10.1136/gutjnl-2023-330389, PMID: 37788894

[B15] Liu J. Li T. Zhang L. Xu A. (2019). The role of hepatitis B surface antigen in nucleos(t)ide analogues cessation among asian patients with chronic hepatitis B: A systematic review. Hepatology 70, 1045–1055. doi: 10.1002/hep.30474, PMID: 30561829

[B16] Liu Z. Yin B. Xu L. Yu Y. Yang Y. Wu X. (2025). Mitochondria-related parameters of lymphocyte subsets can distinguish different disease stages in patients with HBV infection. Sci. Rep. 15, 21008. doi: 10.1038/s41598-025-05922-0, PMID: 40596387 PMC12215614

[B17] Ma L. Han Q. Cheng L. Song H. Qiang R. Xu P. . (2024). Altered mitochondrial mass and low mitochondrial membrane potential of immune cells in patients with HBV infection and correlation with liver inflammation. Front. Immunol. 15, 1477646. doi: 10.3389/fimmu.2024.1477646, PMID: 39650657 PMC11621101

[B18] Mansouri A. Gattolliat C. H. Asselah T. (2018). Mitochondrial dysfunction and signaling in chronic liver diseases. Gastroenterology 155, 629–647. doi: 10.1053/j.gastro.2018.06.083, PMID: 30012333

[B19] Mehrotra A. D’Angelo J. A. Romney-Vanterpool A. Chu T. Bertoletti A. Janssen H. L. A. . (2020). IFN-α Suppresses myeloid cytokine production, impairing IL-12 production and the ability to support T-cell proliferation. J. Infect. Dis. 222, 148–157. doi: 10.1093/infdis/jiaa064, PMID: 32049318

[B20] Schurich A. Pallett L. J. Jajbhay D. Wijngaarden J. Otano I. Gill U. S. . (2016). Distinct metabolic requirements of exhausted and functional virus-specific CD8 T cells in the same host. Cell Rep. 16, 1243–1252. doi: 10.1016/j.celrep.2016.06.078, PMID: 27452473 PMC4977274

[B21] Shao J. Wei L. Wang H. Sun Y. Zhang L. F. Li J. . (2007). Relationship between hepatitis B virus DNA levels and liver histology in patients with chronic hepatitis B. World J. Gastroenterol. 13, 2104–2107. doi: 10.3748/wjg.v13.i14.2104, PMID: 17465456 PMC4319133

[B22] Su Y. Bu F. Zhu Y. Yang L. Wu Q. Zheng Y. . (2024). Hepatitis B virus core protein as a Rab-GAP suppressor driving liver disease progression. Sci. Bull. (Beijing) 69, 2580–2595. doi: 10.1016/j.scib.2024.04.014, PMID: 38670853

[B23] Terrault N. A. Lok A. S. F. McMahon B. J. Chang K. M. Hwang J. P. Jonas M. M. . (2018). Update on prevention, diagnosis, and treatment of chronic hepatitis B: AASLD 2018 hepatitis B guidance. Hepatology 67, 1560–1599. doi: 10.1002/hep.29800, PMID: 29405329 PMC5975958

[B24] Tout I. Loureiro D. Mansouri A. Soumelis V. Boyer N. Asselah T. (2020). Hepatitis B surface antigen seroclearance: Immune mechanisms, clinical impact, importance for drug development. J. Hepatol. 73, 409–422. doi: 10.1016/j.jhep.2020.04.013, PMID: 32333923

[B25] Tsai Y. N. Wu J. L. Tseng C. H. Chen T. H. Wu Y. L. Chen C. C. . (2024). Hepatitis B core-related antigen dynamics and risk of subsequent clinical relapses after nucleos(t)ide analog cessation. Clin. Mol. Hepatol. 30, 98–108. doi: 10.3350/cmh.2023.0194, PMID: 38092551 PMC10776300

[B26] Tseng T. C. Chiang C. Liu C. J. Hong C. M. Su T. H. Yang H. C. . (2023). Low hepatitis B core-related antigen levels correlate higher spontaneous seroclearance of hepatitis B surface antigen in chronic hepatitis B patients with high hepatitis B surface antigen levels. Gastroenterology 164, 669–79.e6. doi: 10.1053/j.gastro.2023.01.005, PMID: 36642151

[B27] van der Windt G. J. Everts B. Chang C. H. Curtis J. D. Freitas T. C. Amiel E. . (2012). Mitochondrial respiratory capacity is a critical regulator of CD8+ T cell memory development. Immunity 36, 68–78. doi: 10.1016/j.immuni.2011.12.007, PMID: 22206904 PMC3269311

[B28] Wang J. Zhang Z. Zhu L. Zhang Q. Zhang S. Pan Y. . (2024). Association of hepatitis B core antibody level and hepatitis B surface antigen clearance in HBeAg-negative patients with chronic hepatitis B. Virulence 15, 2404965. doi: 10.1080/21505594.2024.2404965, PMID: 39317345 PMC11423664

[B29] Winkler F. Hipp A. V. Ramirez C. Martin B. Villa M. Neuwirt E. . (2023). Enolase represents a metabolic checkpoint controlling the differential exhaustion programmes of hepatitis virus-specific CD8(+) T cells. Gut 72, 1971–1984. doi: 10.1136/gutjnl-2022-328734, PMID: 37541771 PMC10511960

[B30] Xu Y. Liu Y. Cao Z. Wang L. Li Z. Sheng Z. . (2019). Comparison of FibroTouch and FibroScan for staging fibrosis in chronic liver disease: Single-center prospective study. Dig Liver Dis. 51, 1323–1329. doi: 10.1016/j.dld.2019.02.009, PMID: 30928419

[B31] Ye B. Liu X. Li X. Kong H. Tian L. Chen Y. (2015). T-cell exhaustion in chronic hepatitis B infection: current knowledge and clinical significance. Cell Death Dis. 6, e1694. doi: 10.1038/cddis.2015.42, PMID: 25789969 PMC4385920

[B32] Yin G. Q. Chen K. P. Gu X. C. (2022). Heterogeneity of immune control in chronic hepatitis B virus infection: Clinical implications on immunity with interferon-α treatment and retreatment. World J. Gastroenterol. 28, 5784–5800. doi: 10.3748/wjg.v28.i40.5784, PMID: 36353205 PMC9639659

[B33] You H. Wang F.-S. Li T. Xu X. Sun Y. Nan Y. . (2023). Guidelines for the prevention and treatment of chronic hepatitis B (Version 2022). Infect. Dis. Immun. 03, 145–162. doi: 10.14218/JCTH.2023.00320, PMID: 37719965 PMC10500285

[B34] Zhao Q. Liu H. Tang L. Wang F. Tolufashe G. Chang J. . (2024). Mechanism of interferon alpha therapy for chronic hepatitis B and potential approaches to improve its therapeutic efficacy. Antiviral Res. 221, 105782. doi: 10.1016/j.antiviral.2023.105782, PMID: 38110058

[B35] Zhou R. R. Song Y. H. Xu C. Y. Zhang Y. Y. Wu X. W. Zhang L. . (2024). Altered counts and mitochondrial mass of peripheral blood leucocytes in patients with chronic hepatitis B virus infection. J. Cell Mol. Med. 28, e18440. doi: 10.1111/jcmm.18440, PMID: 38890792 PMC11187856

[B36] Zoulim F. (2004). Mechanism of viral persistence and resistance to nucleoside and nucleotide analogs in chronic hepatitis B virus infection. Antiviral Res. 64, 1–15. doi: 10.1016/j.antiviral.2004.07.003, PMID: 15451174

