# Supplementary figures and images for: Mitochondrial metabolic remodeling predicts therapeutic response to PegIFN-α in chronic hepatitis B
Source: Front Cell Infect Microbiol. 2026 Jan 14;15:1719456. doi: 10.3389/fcimb.2025.1719456 (PMC12847435; doi:10.3389/fcimb.2025.1719456)

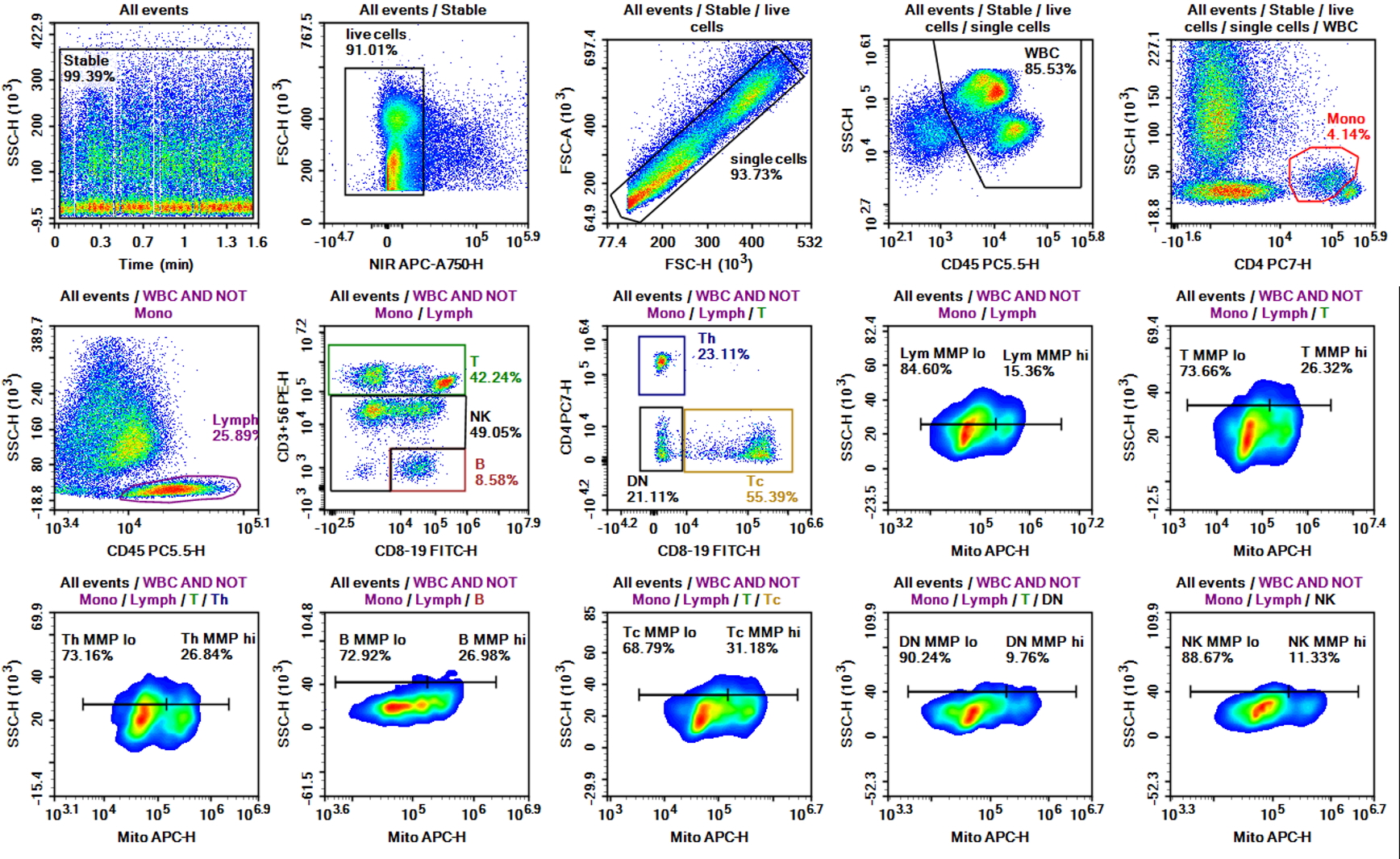

Supplement: Supplementary Figure 1 — Manual gating strategy. Figure shows the gating strategy for lymphocyte subsets and mitochondrial parameters (MM and MMPlow%) by flow cytometry. The CD3 antibody is in the same lane (PE) as the CD56 antibody but has a different level of fluorescence intensity, which allows us to specifically recognize cell populations. The same forms of CD19 and CD8 are the same. The main population of cells can be identified based on different expressions, and the aim is to use fewer channels to achieve multi-parameter detection (patent number: CN 112098646 B). The Mito APC-H channel shows the mitochondrial MMP clustering of the corresponding cell population. [file Image1.jpeg]
